# On the Speculum Vaginæ

**Published:** 1859-04

**Authors:** John P. Mettauer

**Affiliations:** Virginia


### ON THE SPECULUM VAGINÆ.

BY JOHN P. METTAUER, M.D., LL.D., OF VIRGINIA.

The employment of this instrument, of late years, in the exploration and treatment of uterine affections, has become almost as common as the stethoscope and percussion in the diseases of the thoracic organs. Even inexperienced practitioners, who have barely laid aside the swathings of their pupilage, presume to employ it, and speak authoritatively of the mode of applying it, as well as of the diseases demanding its use. They seem to regard the operation as a thing of little importance, as far as female delicacy is concerned, and to believe that poor woman should submit to it, even if a disease of the uterus is only suspected to exist, that might possibly render the speculum necessary hereafter.

Every enlightened and humane physician will concede that a necessity will sometimes arise for the employment of the speculum, as well as other modes of exploration, repulsive to female delicacy. In such cases a sacrifice of delicacy becomes a duty, and sensible women unhesitatingly submit to its wise and sacred behests.

The writer has undertaken this communication for the purpose of showing that the speculum, in the investigation and treatment of uterine diseases, has been needlessly employed, and its value, as a means of diagnosis, greatly abused. That the instrument is entirely unnecessary in a large majority of uterine diseases, the writer's experience abundantly testifies. His experience with the speculum, too, has long since satisfied him that the evidence furnished by it is often unsatisfactory, and not to be relied on; nay, in some instances, it is actually deceptive, by reason of the changes caused in the state of the os and cervix uteri, by the pressure of the instrument on them. It has frequently been the case, in the hands of the writer, that the pressure of the speculum has so changed the color and presenting surface of those parts, as actually to defeat the objects of the examination; and such will often be the case in engorgement, the deviation of position, internal ulceration, and very frequently, of ulceration of the os itself, no matter how carefully and skillfully used, it affords little, if any, information of a reliable and useful nature. Even when the three or four-bladed instrument is employed, the operation and results will be obnoxious to these objections in a great degree, and they are the only reliable forms of vagino-uterine speculums in displaying the parts to be examined, and are also more readily and easily introduced; yet, little difficulty will be encountered in the use of any of the speculums now in use, even with a mere novice, who has carefully studied and learned the form, course and depth of the vagina, the highly wrought and faithful account of such difficulties, published in the *Monthly Stethoscope and Medical Reporter*, No. 2, Vol. II., for 1857, to the contrary notwithstanding.

It is not pretended that the speculum is useless, or absolutely unnecessary in vaginal and uterine diseases. Far otherwise—as the writer has employed it in those diseases, in some instances, with the best results. It is to the officious and indiscriminate use of it that he objects, and to the exclusion and neglect of the more reliable and delicate mode of examination by the "toucher."

The speculum has not found general favor in France, although more employed in that country. At the head of its opponents there, the name of the distinguished Velpeau stands conspicuous; and it is matter of gratulation to the writer to find his views supported by such high authority; yet he entertained these views and carried them out in practice years before he was aware that Velpeau had expressed similar opinions and objections.

It is probable that the physicians of this country and France, more generally and indiscriminately employ the speculum than any others in the civilized world; and it is probable, also, that the taste for using it is due, in a degree, if not wholly, to the cliniques, as well as to the hospital practice connected with the medical schools of those countries where female delicacy and exposure are regarded with little concern, as the subjects of the use of the speculum are derived from the most degraded classes of society, with whom modesty is only known by name. In many instances, the writer has met with women laboring under organic disease of the uterus, who declared to him that they would sooncr take their chance to live and die with the disease, than submit to the use of the speculum; and all are more or less opposed to it, even those who finally submit to its employment. Really, it is not to be wondered at, that a modest, delicate woman should feel unwilling to submit her person to such a revolting exposure; and the writer candidly owns that he has never yet applied the speculum, or even examined by toucher, without being more or less abashed and disconcerted, by reason of the exposure the operation necessarily imposes on females. Even the ordinary modes of investigation by question and answer, often greatly shock a modest female, and in a degree, in some instances, embarrass the diagnosis of her diseases.

When organic disease of the uterus exists, and the rational symptoms fail in furnishing the requisite amount of information necessary to form a satisfactory diagnosis, nearly every intelligent woman will consent to a physical examination, if made sensible of the necessity for it, especially if the proposition to do so is delicately presented; and such being the case, it is the duty of the physician, as far as is consistent with safety, to save his female patients all needless shock of feeling from delicate questions or personal exposure.

Entertaining such views of this delicate subject, the writer, some ten years since, directed his attention to the investigation of organic diseases of the uterus, guided by the toucher, chiefly; and, after repeated trials, affording ample experience, he unhesitatingly states that the information it furnishes is far more reliable and satisfactory than that derived from any form of speculum, in determining as to the existence and nature of such diseases. In numerous instances, during the time above stated, he has tested the correctness of his diagnosis in uterine diseases, guided by the taxis. Most of the examples presented ulceration of the os, but in many cases the cervix was also implicated more or less extensively. Ten of them exhibited the os patulous, exceeding in size a Spanish dollar, and deeply ulcerated, the cervix indurated considerably beyond the interior boundary of the corresponding border of the ulcer, and the general health greatly impaired.

After carefully examining into the condition of the os and cervix uteri by the toucher, he was enabled to detect ulceration with great certainty, as well as induration, engorgement, and all of the deviations of position.

An ulcerated os uteri presents to the experienced touch the same feel as an ulcer on the exterior of the body; and an accompanying induration of the surrounding parts is a very common attendant of such ulceration, as it is also of many external ulcers. Induration of the cervix, however, is decidedly more apt to accompany intra-cervical ulceration; and as it is uniformly met with in such ulceration of the cervix, clearly ascertained to exist, as well as frequently in ulceration of the os likewise, it may safely be inferred that it represents ulceration in all those cases in which the cervix is inaccessible to the touch, when indurated, without ulceration of the os.

In deciding as to the existence of induration of the os or cervix uteri, the speculum is absolutely useless. Even in ulceration, the information it imparts is unsatisfactory and unreliable. In engorgement and inflammation, it furnishes no information that is not derivable from the toucher, elucidative of those conditions, and is far more offensive to the feelings of a delicate woman than the investigation by the taxis.

The discharge, said to be characteristic of, and peculiar to, ulceration of the os and cervix, is not by any means constant in appearance, nor does it furnish conclusive evidence in all cases that ulceration does exist when met with. If present, and just issuing from the os uteri, either in its semi-fluid or ropy condition, the speculum, if then applied, would only prove that the morbid secretion unequivocally proceeded from the os uteri. The discharge of this diseased product externally, however, affords as satisfactory evidence of ulceration of the os uteri, as if actually seen escaping from the uterine cavity, because its characters are sufficiently marked to remove all doubts of its identity.

Although furnishing pretty satisfactory evidence of the existence of organic diseases of the uterus, of itself, the revelations of the toucher should invariably be taken in connection with the other symptoms usually met with in such diseases, in forming a diagnosis. The ulcerated os and cervix, when accessible to the touch; the induration; the peculiar discharge; pelvic and dorsal pains; inability to stand at a long time; frequently, abdominal pains; disordered digestion; nervousness; depression of spirits, and the peculiar desponding expression of countenance termed "facies uterine," when taken together, leave little room to doubt as to the existence of ulceration of the os and cervix uteri.

The speculum will be demanded in those cases in which the os uteri cannot be reached by the finger, as then no other reliable plan could be adopted for exploring, and treating such examples. Fortunately, these latter instances are rarely to be met with, as the writer has only witnessed two out of over a hundred cases treated by him in ten years. It will also be required in scirrhous uteri, when the indurated cervix is to be excised, and when adhesions between the os or cervix and vagina exist. And it will be indispensable in cauterizing the uterus with the incandescent iron, and in leeching or scarifying the organ.

For the purpose of cauterizing the os and cervix, the writer employs the nitrate of silver, and the acid nitrate of mercury, conveyed to the parts, concealed by a canula directed by the index finger of the right hand; and the operation should be repeated once in three or four days, or after longer intervals, if the previous operation is followed by prolonged bleeding, until the cure is perfected. The nitrate of silver is best adapted to the mild or slight examples of ulceration; while the acid nitrate of mercury should be used when the ulcers are deep and extensive, and especially if the cervix is decidedly implicated. It is best, however, to begin the treatment with the nitrate of silver; and if amelioration seems tardy, then to employ the acid nitrate of mercury in alternation with the caustic silver.

The position most convenient to the operator for examination, as well as for the application of remedies, is on the left side, with the thighs flexed on the trunk, and the legs on the thighs. The person should invariably be covered, and the nates placed near the border of a bed. In this posture, the parts can generally be reached and examined with the index finger of the right hand with entire convenience; and it is also best for the application of the speculum, as well as the cauterizing agents employed through it.

The first trials, in the use of the caustic, upon the plan advocated in this paper, will, in all probability, be attended with some difficulty; but gentle efforts, repeated again and again deliberately, will soon impart the requisite dexterity of manipulation to insure success; and, after learning how to apply the remedy, the ease with which it can be done will astonish both patient and physician.

A crayon formed of the nitrate of silver, or the stick itself, may be used, applied as already intimated; and, for the application of the acid nitrate of mercury, a short, full camel's-hair brush, or mop, saturated with the undiluted solution, answers best. The canula should be fully ten inches in length, of proper calibre to contain the crayon, or mop, and open at both ends, so as to allow the handle of the crayon to project sufficiently beyond the free, or outer extremity, so as to be held and wielded by the operator's left hand; and it may be formed of silver or glass—the latter material the writer employs, and decidedly prefers.

To guard against vaginal irritation, from accidental diffusion of either of the caustics over its surface, after being applied to the uterus, a weak solution of common salt should be invariably injected into the vagina immediately after any cauterization—using for the purpose a female glass syringe—taking care at the same time that this saline solution is effectually applied to the upper portion of the passage immediately around the cervix uteri. After this the vagina may be abluted daily with simple water, or mucilaginous infusions, such as slippery elm or flaxseed teas, applied tepid or cool, as may be preferred by females. The saline wash may also be used tepid to cool, according to the fancy of different patients.

The bowels should be kept in a soluble, easy condition, using for the purpose, when necessary, mild aperients, especially gentle aloetic preparations. When induration of the cervix exists, and if the habit is anæmic, the iodide of iron will be proper. If anæmia, without induration, is present, and more especially should there be nervous debility, and marked depression of spirits, frequently tending to deep despondency, the phosphate of iron will be indicated. It will sometimes be necessary to resort to vegetable tonics in these cases; and in many instances nothing answers better than good porter. The cold infusion of wild cherry bark (prun. virgin.) will very often supersede all other vegetable tonics; and the cases most likely to be benefited by it are those attended with undue nervousness, as well as debility. When the liver is torpid, and the bowels refuse to respond to the action of aperients, the nitro-muriatic acid mixture will be found signally beneficial. The diet should invariably be simple, and moderately nutritious.

It will greatly promote recovery, to require patients to remain in bed, or in a recumbent posture, during treatment; and, for months after recovery, every species of traveling will be hurtful. The utmost care should be taken to guard patients against exposure to variable temperature. Catarrhal disturbances invariably aggravate uterine diseases of every kind, and in none do they prove more hurtful than in ulceration and induration of the os and cervix.—*Virginia Medical Journal.*

